# Cardiologists’ Knowledge and Perception towards American Heart Association Guidelines of Cardiopulmonary Resuscitation; a Letter to Editor 

**DOI:** 10.22037/aaem.v9i1.1053

**Published:** 2021-01-02

**Authors:** Sajjad Ali, Annam Zahid, Syed Zahid Jamal, Samahir Tariq Khan, Nisha Lohana, Raahim Ahmed, Nobia Mehdi

**Affiliations:** 1 Internal Medicine Department, Ziauddin Medical University, Karachi, Pakistan.; 2 National Institute of Cardiovascular Diseases, Karachi, Pakistan.

**Keywords:** Cardiopulmonary Resuscitation, Cardiologists, Heart Arrest, Pakistan, Advanced Cardiac Life Support

**Dear editor**

Sudden cardiac arrests (SCA) pose massive threats to millions of lives worldwide. Latest statistics report an annual death count of approximately 17.9 million for cardiovascular diseases (1). SCA has caused 15% of these deaths (2) and is considered a major threat in both out-of-hospital and in-hospital settings. Early identification and prompt cardiopulmonary resuscitation (CPR) is essential to increase the survival rate of SCA patients from about 50% to 70% (3). To accomplish this increment, it is cardinal for each physician to be versed with the protocols of basic life support (BLS) and advanced cardiac life support (ACLS). 

Inadequacy with reference to both knowledge and awareness of guidelines has been witnessed in various low-income, lower-middle income, and middle-income countries such as Greece, India, and Nigeria (4-6). A study showed a significant lack of knowledge and practical approach regarding emergency cardiovascular care (ECC), among junior healthcare professionals (7). Once training has been undertaken, it is indicated that skills may decline in a year, especially if not frequently performed or reviewed (8). Several studies from Pakistan have identified similar knowledge deficits regarding resuscitation guidelines among healthcare professionals (9, 10); however, no such research has been solely aimed at cardiologists. We, surveyed cardiologists’ theoretical knowledge of resuscitation guidelines, with an additional set of questions directed to test presumptive correlational factors, in National Institute of Cardiovascular Diseases (NICVD), Pakistan.

Results showed that out of the 215 studied cardiologists, only 162 (75.3%) cases were certified and had high mean test scores. Surprisingly, the mean percentage of correctly marked theoretical questions was only 50.9 (ranging from 25.0 to 75.0). 212 participants believed that ACLS courses should be taken by students during medical school and 203 thought that annual retraining or refresher training of all healthcare professionals was necessary for having up-to-date knowledge on the AHA guidelines. The length of time practicing in the sub-specialty had a negative correlation with knowledge scores (p = 0.039), as participants with less experience had the highest average score (52.03%). Most respondents felt confident in their current knowledge of the AHA guideline displaying an average knowledge score of 50.92%. The results of this study concerning mean knowledge scores of participants regarding AHA-CPR guidelines based on different baseline characteristics are illustrated in table 1. Table 2 shows the perception of participants regarding AHA-CPR guidelines’ education and training.

Despite proven effectiveness of training courses, physicians may not fully comprehend the significance of recurring courses. The recommendation for refresher training, as proposed by previous study findings, is biannual i.e. every 6 months (11). Even though optimal timelines for recommended retraining is yet unknown, it is deduced that regular retraining is required to gain proper resuscitation skills.  

Another contributing element of resuscitation skills was lack of early training and exposure, especially during graduate school. Medical school curriculum is largely based on long-term patient care, possibly overlooking acquaintance with emergency care approaches, rendering them unimportant in the early years. ACLS courses could be included under Continued Medical Education (CME), in our opinion. CME being included under CME makes it necessary for practitioners to undergo retraining to maintain licensure among other entitlements. Hospitals or medical councils, can subsidize the financial burden of these costly ACLS courses in conjunction with CME. With reference to the disparities in healthcare, primarily caused by low national budgets dispensed for health-related causes, cardiologists in Low and Low-middle income countries should be monetarily assisted to gain life-saving skills for both in-hospital and out-of-hospital emergencies. 

It could be concluded that, theoretical knowledge of CPR guidelines among Pakistani cardiologists remains unsatisfactory. Regular refresher and/or retraining courses of ACLS might improve the quality of CPR techniques implemented and hence, downsize the global health burden caused by SCAs. We further believe that BLS and ACLS courses should be integrated into the curriculums at all medical schools in Pakistan. 

**Table 1:** Comparison of mean knowledge scores of participants regarding AHA-CPR guidelines based on different baseline characteristics

**Characteristics **	**Number**	Score*	**P **
Specialty			
Invasive	87	48.63 ± 11.53	0.033
Non-invasive	128	52.39 ± 10.37	
**Gender**			
Male	171	50.54 ± 10.80	0.345
Female	44	52.13 ± 11.74	
**Positions**			
Attending/consultant	49	48.47 ± 11.72	0.105
Resident	166	51.58 ± 10.69	
**Institution**			
University hospital	92	50.06 ± 11.13	0.215
Educational research hospital	123	51.47 ± 10.88	
**Years in specialty **			
0 - 5	175	52.03 ± 10.76	0.039*
6 - 10	30	45.41 ± 11.12	
11 - 20	6	45.83 ± 9.41	
21 - 30	3	50.00 ± 12.50	
> 30	1	50.87 ± 0.00	
**Updated with AHA guidelines?**			
Yes	208	50.96 ± 11.08	0.438
No	7	48.21 ± 7.83	
**Attendance in an ACLS course**			
Yes	179	51.46 ± 10.96	0.053
No	36	47.91 ± 10.77	
**Time from last ACLS course (years)**			
< 1	82	48.85 ± 10.26	0.095
1 – 5	123	52.23 ± 11.54	
6 – 10	10	50.62 ± 7.48	
**ACLS certification?**			
Yes	162	51.81 ± 11.05	0.014*
No	53	47.99 ± 10.38	

*: Measures are presented as mean ± standard deviation. ACLS: Advanced Cardiac Life Supports; AHA: American Heart Association; CPR: cardiopulmonary resuscitation.

**Table 2:** Perception of participants regarding AHA-CPR guidelines’ education and training

**Perception **	**Number** (%)
**Would you recommend medical students to take an ACLS course?**	
Yes	212 (98.6)
No	1 (0.5)
Maybe	2 (0.9
**Do you think healthcare professionals should update their knowledge on AHA guidelines as per the annual update release?**	
Yes	203 (94.4)
No	4 (19)
Maybe	8 (3.7)
**Do you think ACLS courses should be re-evaluated frequently? **	
Yes	177 (82.3)
No	8 (3.7)
Maybe	30 (14.0)
**Do you think you are confident in saving a life with your AHA guidelines knowledge? **	
Yes	209 (97.2)
No	2 (0.9)
Maybe	4 (1.9)
**Is it important for healthcare professionals from all disciplines to attend ACLS courses? **	
Yes	209 (97.2)
No	2 (0.9)
Maybe	4 (1.9)

Data are presented as number and percentage. ACLS: Advanced Cardiac Life Support; AHA: American Heart Association; CPR: cardiopulmonary resuscitation.

## Conflict of interests

The authors declared no potential conflicts of interest with respect to the research, authorship, and/or publication of this article.

## Authors' contributions

**Sajjad Ali**: Conceptualization, Supervision, Project administration, Writing - Original Draft, Data Curation, Formal analysis**. Annam Zahid**: Writing - Original Draft, Writing - Review & Editing, Data Curation, Resources, Investigation.** Syed Zahid Jamal**: Supervision, Project administration. **Samahir Tariq Khan**: Writing - Original Draft, Writing - Review & Editing, Investigation. **Nisha Lohana**: Writing - Original Draft, Data Curation. **Raahim Ahmed**: Writing - Original Draft, Methodology. **Nobia Mehdi**: Validation, Supervision
